# microRNA-494 and ATF3 the targets of onconase(?)

**DOI:** 10.18632/oncotarget.13799

**Published:** 2016-12-05

**Authors:** Zbigniew Darzynkiewicz

**Affiliations:** Department of Pathology, New York Medical College, Valhalla, USA

**Keywords:** anticancer drugs, apoptosis, RNA interference, ranpirnase, nuclear factor kappa B

Onconase (Onc; also named ranpirnase), a 12 kD ribonuclease from oocytes of northern leopard frogs (*Rana pipiens*), is cytostatic and cytotoxic to variety tumor cell lines, inhibits growth of tumors in model animals and advanced to clinical trials as anticancer and antiviral drug [1]. Although its cytostatic and cytotoxic properties were recognized nearly three decades ago [2] mechanism of action is still elusive. The anticancer properties of Onc require its ribonucleolytic activity sustained by resistance to ribonuclease inhibitor protein that is present in cytosol as well as to its remarkable conformational stability. However, it is unclear which RNA species are preferential Onc targets whose destruction may explain anticancer specificity of this RNase. The initial observation that rRNA and tRNA become degraded in Onc-treated cells led the authors to propose that suppression of translation is the primary cause of its activity. However subsequent observations of cells sensitivity to Onc, its effect on the cell cycle, and complexity of other effects that lead to cell death were incompatible with this notion. In fact the evidence was accumulating that different signaling pathways become activated and diverse genes were up- and down-regulated [3].

In this issue of Oncotarget Vert *et al*., [4] report on their attempts to identify the Onc regulated genes that could explain the cytotoxic and anticancer properties of this RNase. Using the microarray-derived transcriptional profiling, confirmed by RT-qPCR, they noticed that among the up-regulated genes the most conspicuous is activating transcription factor 3 (ATF3). This was the case for both ovarian cancer cell lines analyzed by the authors. Together with up-regulation of several genes downstream of ATF3, the character of cell cycle changes and other effects leading to cell apoptosis, the data suggest that activation of ATF3 is the key event responsible for cytotoxicity of Onc and for its specificity to cancer cells. Also the prior studies [3] exploring the up- and down-regulation of different genes by Onc in malignant mesothelioma cell lines, conform to these findings. The reported data are also congruent with the observed antiviral properties of Onc in as much as this transcription factor can be implicated in suppressing viral genome replication, keeping virus latency or preventing viral oncogenesis [4].

The findings that ATF3 is the Onc target are in accordance with earlier reports that activation of ATF3 by other than Onc means triggers signaling pathways that lead to apoptosis in different types of cancers. Of particular interest are observations that overexpression of ATF3 increases cell sensitivity to variety of anticancer drugs. Thus, there is a remarkable correspondence between the mentioned above effects of ATF3 and Onc as factors increasing sensitivity of cancer cells to other treatments. Namely, dozens of papers describing ability of Onc to sensitize cancer cells to different anticancer drugs are recorded in PubMed. In some of these publications Onc is proposed to be used as an adjunctive therapy to amplify effectiveness of the primary treatment. The suppression of NF-kB by Onc was advanced to explain its ability to sensitize cancer cells *via* decreasing resistance to apoptosis [5]. The involvement of NF-κB as one of the mechanisms by which Onc is increasing sensitivity of cancer cells to other drugs is consistent with up-regulation of ATF3 as the central event affecting transcription of the genome of treated cancer cells [4].

The noncoding RNA that provides regulation of genes activity via RNAi appears to be the potential primary target of this Onc. It was already demonstrated that Onc is able to attack siRNA within the cell and activate the siRNA-suppressed gene [6]. It is possible therefore that activation of ATF3 by Onc is mediated by targeting siRNA or other RNAi mechanisms that otherwise suppress activity of this gene. Thus far the only candidate that can be found in the literature is microRNA-494 which by binding to the 3′UTR of ATF3 directly suppresses transcription of this factor. In mice its overexpression significantly attenuates the level of ATF3 [7]. Perusal of the literature also shows that modulation of microRNA-494 has profound effects on cancer cells proliferation and sensitivity to apoptosis. The primary potential target of Onc, at present, appears to be the microRNA-494.

Activation of ATF3 *via* RNAi by Onc does not preclude the eventuality that other RNA species are targets of Onc as well. Both rRNA and tRNA have been shown degraded by Onc. Our early data on human submaxillary carcinoma A-253 and colon adenocarcinoma Colo 320 CM cells indicated that in addition to reduced clonogenicity the size of individual cells as well of cell clones was substantially smaller in the Onc treated cultures [2]. Also the Onc treated cells had diminished RNA content. All this suggests that growth rate of the surviving cells was reduced. This may be expected if rate of translation is suppressed by partial targeting rRNA and/or tRNA. Upon entrance of Onc into the cell, thus, equilibrium may develop between different rates of degradation of different RNA species e.g. due to differences of their accessibility to this RNase. As shown [4] however regardless of the cell type the dominant effect in terms of a loss of cell viability appears to be activation of transcription of ATF3. The data in the literature point out that ATF3 selectivity may provide its anticancer properties, perhaps across different cancer types. The antiviral properties of Onc are also compatible with this mechanism when different RNA substrates including viral genomes and host-cell nucleic acids used for viral replication all are accessible to Onc.

## References

[1] Ardelt W (2009). Eur J Pharmacol.

[2] Darzynkiewicz Z (1988). Cell Tissue Kinet.

[3] Altomare DA (2010). BMC Cancer.

[4] Vert A (2016). Oncotarget.

[5] Tsai SY (2004). Int J Oncol.

[6] Zhao H (2008). Cell Cycle.

[7] Lan YF (2012). J Am Soc Nephrol.

